# Artificial Intelligence Algorithm Supporting the Diagnosis of Developmental Dysplasia of the Hip: Automated Ultrasound Image Segmentation

**DOI:** 10.3390/jcm14176332

**Published:** 2025-09-08

**Authors:** Łukasz Pulik, Paweł Czech, Jadwiga Kaliszewska, Bartłomiej Mulewicz, Maciej Pykosz, Joanna Wiszniewska, Paweł Łęgosz

**Affiliations:** 1 Department of Orthopedics and Traumatology, Medical University of Warsaw, Lindley 4 Str., 02-005 Warsaw, Poland; pawel.legosz@wum.edu.pl; 2 Pentacomp Systemy Informatyczne S.A., Aleje Jerozolimskie 179 Str., 02-222 Warsaw, Poland; pawel.czech@pentacomp.pl (P.C.); bartek.mulewicz@pentacomp.pl (B.M.); maciej.pykosz@pentacomp.pl (M.P.); joanna.wiszniewska@pentacomp.pl (J.W.); 3 Gustav—Children’s Clinic, Białej Floty 2 Str., 02-654 Warsaw, Poland; j.kaliszewska@gustav.pl

**Keywords:** developmental dysplasia of the hip, Graf method, hip ultrasound, artificial intelligence, image segmentation, deep neural networks

## Abstract

**Background**: Developmental dysplasia of the hip (DDH), if not treated, can lead to osteoarthritis and disability. Ultrasound (US) is a primary screening method for the detection of DDH, but its interpretation remains highly operator-dependent. We propose a supervised machine learning (ML) image segmentation model for the automated recognition of anatomical structures in hip US images. **Methods**: We conducted a retrospective observational analysis based on a dataset of 10,767 hip US images from 311 patients. All images were annotated for eight key structures according to the Graf method and split into training (75.0%), validation (9.5%), and test (15.5%) sets. Model performance was assessed using the Intersection over Union (IoU) and Dice Similarity Coefficient (DSC). **Results**: The best-performing model was based on the SegNeXt architecture with an MSCAN_L backbone. The model achieved high segmentation accuracy (IoU; DSC) for chondro-osseous border (0.632; 0.774), femoral head (0.916; 0.956), labrum (0.625; 0.769), cartilaginous (0.672; 0.804), and bony roof (0.725; 0.841). The average Euclidean distance for point-based landmarks (bony rim and lower limb) was 4.8 and 4.5 pixels, respectively, and the baseline deflection angle was 1.7 degrees. **Conclusions**: This ML-based approach demonstrates promising accuracy and may enhance the reliability and accessibility of US-based DDH screening. Future applications could integrate real-time angle measurement and automated classification to support clinical decision-making.

## 1. Introduction

Developmental dysplasia of the hip (DDH) is the most common musculoskeletal disorder in newborns [1]. It affects approximately 1.4% (95% CI: 0.86 to 2.28) of the world’s population, but the incidence of DDH can vary from 0.5% to 30% depending on geographical and ethnic origin [2]. The term DDH reflects a broad spectrum of abnormalities, from mild acetabular deficiency to severe cases with dislocation of the femoral head. In less severe cases, DDH can remain clinically silent in children due to the limited sensitivity and specificity of clinical examination findings, such as hip abduction difference, asymmetric skin creases, or hip instability tests. If not treated, it often leads to the development of hip osteoarthritis (OA) in young adults, which may require total hip arthroplasty (THA) [3]. Up to 40% of patients who undergo THA under 50 years of age have radiological signs of DDH [4]. In severe DDH, symptoms usually appear at birth, including hip instability, shortening of the limb, and limited abduction of the affected hip. In children, we can observe gait problems or a delay in the onset of walking [5]. Conservative treatment (bracing) has the highest success rate if DDH is diagnosed during the first 6 weeks of life. When the diagnosis is delayed or incorrect, surgery may be necessary [6].

Before the introduction of hip ultrasound (US) by Reinhard Graf (1980s), the diagnosis of DDH was based on clinical examination and radiographic imaging. As mentioned above, clinical examination has only limited efficacy in the detection of DDH, and radiograph-based diagnosis is only possible after 4 to 6 months of age, which is too late for efficient conservative treatment [7]. Today, ultrasound is the gold standard in the diagnosis and monitoring of DDH treatment [8]. Graf’s method evaluates acetabulum development by measuring the α angle on standard plane US hip scans. Depending on the *α* angle, hips are classified (I-IV), and the *β* angle determines the hip subtype. Graf’s method is characterized by high sensitivity and specificity in DDH detection, respectively, at 93% (95% CI 0.57–0.99) and 97% (95% CI 0.86–0.99) [9]. However, many errors in the application of the method are reported in the literature, and the use of the Graf method requires experience and strict adherence to the usability checklists [10]. In Europe, many countries have adopted universal ultrasound screening, resulting in a high demand for specialists in the field of DDH diagnosis [8].

The solution for the large volume of US hip scans required could be the recently developed ML-based algorithms. They can improve the reliability of the diagnosis, which is often compromised by the relatively poor quality of the images due to the presence of noise and acquisition errors. They can provide a “second opinion” in diagnosis by standardizing image interpretation and reducing operator-dependent errors [11]. Various diagnostic applications of AI in musculoskeletal ultrasound already include the detection, diagnosis, and prognosis of diseases [12]. The precision of such an application of AI in hip US could equal or even outperform that of humans. Some AI-based systems to detect DDH have already been approved by the FDA [13]. However, none of the systems are fully based on the widely accepted Graf rules of image acquisition. In our study, we present a system for automated identification of anatomical structures and landmarks proposed by Graf in two-dimensional (2D) US images. Subsequently, this system could be used in clinical applications to calculate in real time the α and β angles and suggest the possible diagnosis of DDH.

The purpose of this study is to address the growing need for reliable, high-volume ultrasound screening of infant hips by introducing a machine learning-based system that standardizes image interpretation. Current AI tools for DDH detection are limited, as none are fully aligned with the widely accepted Graf methodology, which remains the clinical gold standard. By developing an automated solution capable of identifying anatomical structures and landmarks required by Graf, this approach has the potential to reduce operator dependency, improve diagnostic consistency, and facilitate large-scale, efficient screening for DDH in future applications.

## 2. Materials and Methods

### 2.1. Study Materials

This was a retrospective cross-sectional observational study. The material for image annotation was collected at a private orthopedic clinic during universal screening US scans (4–6 weeks of life) and follow-up visits. All examinations were performed according to the Graf method by six different orthopedic surgeons trained in this method. The data were acquired in the period between January 2022 and December 2023.

The sample material comes from Mindray USG DC-60S (Shenzhen Mindray Bio-Medical Electronics Co., Ltd., Shenzhen, China), and the settings are individually adjusted by each examiner. Videos (DICOM files) were stored on the PACS server (Mini PACS server HP ProLiant DL20 G10 (Hewlett Packard Enterprise, Houston, TX, USA) with processor: E-2224, RAM: 16 GB) equipped with Mini mEdivum Electronic Ultrasound Examination Archiving System (mEdivum Sp. z o.o., Warsaw, Poland). All data on DICOM files were anonymized using dedicated automated tool.

**Figure 1:**
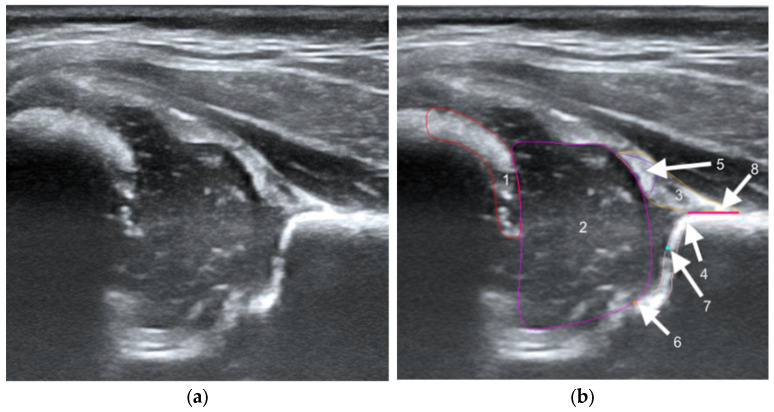
Ultrasound image of the hip in Graf standard plane (**a**) and labeling of anatomical structures (**b**). The labeled diagram (**b**) includes 5 anatomical structures, i.e., chondro-osseous border (red-1), femoral head (purple-2), cartilaginous roof (yellow-3), bony roof (beige-4), and labrum (light purple-5). Other landmarks include lower limb (orange-6), bony rim (light blue-7), and baseline (pink-8).

The structures were annotated by three independent orthopedic surgeons trained in the Graf method who had participated in a Graf-accredited course. The contours of the anatomical structures in the US images were marked using points along the edges of the target anatomy by dedicated software on selected frames. In the annotation process, a maximum of 25 frames from each video were selected by the reporting physician as the best. Annotating physicians selected structures for marking from a drop-down list among the objects on Checklists I and II according to Prof. Graf, necessary for the assessment of hip joints for DDH (Figure 1). Frames were selected for annotation only when it was possible to mark 5 or more objects. Furthermore, at least one selected frame in each video was classified as diagnostic, according to the Graf methodology (standard plane) [14].

To minimize potential sources of bias, several measures were undertaken. One of the primary ways to reduce the risk of error involves real-time automated feedback. The annotation tool identifies common errors, such as overlap or incorrect region selection in ultrasound images. To reduce subjectivity, each annotated frame was independently reviewed by two physicians (cross-check). The professionals were trained not only to use the tool, but also to learn from mistakes made in previous batches of annotations. This involved learning from the feedback of independent reviewers and documenting common errors in the form of a shared knowledge base. The study size was determined pragmatically by including all available scans; this dataset is larger than in most previous AI-DDH studies.

### 2.2. Artificial Intelligence Model

In our approach, the AI model is trained using supervised learning, which usually allows for the best result, but is very demanding regarding the data gathering and labeling process. The model is presented with a US image from a hip scan and returns a segmentation mask with key anatomical structures that contains five structures and two critical points, marked as squares with a size of 5 pixels and a baseline marked as a segment of 3-pixel thickness. Deep neural networks with gradient descent optimization were used. The specifications of the model architectures are described in a later section.

Two approaches to segmentation were tested. In the first approach (Model-5), data for 5 structures were fed into a segmentation model based on convolutional layers and attention mechanisms. In the second approach (Model-8), the data for five structures were augmented with two points (lower limb and bony rim) and the baseline. In both models, five different architectures were tested, and the best one was selected. We tested the following: SegFormer [15], OCRNet [2], U-Net [16], U-HRNet [17], and SegNeXt [18] (Supplementary Table S1). For each architecture, there were many training settings (described in Section 3). Among the many architectures and settings, the one with the best Intersection over Union (IoU) was used.

**Figure 2:**
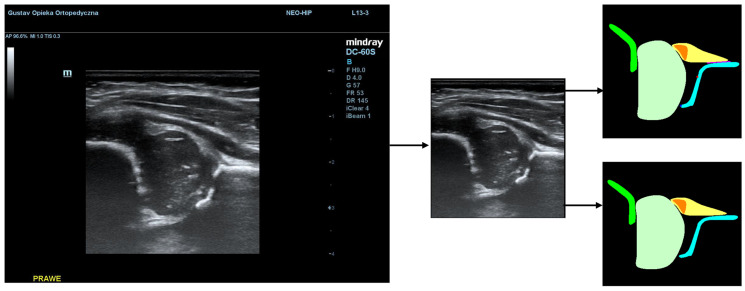
Result of pre-processing: extracting the USG area from the image and preparing masks for Model-5 (**lower picture**) and Model-8 (**upper picture**).

Additionally, during the model training process, the PaddleSeg library uses various pre-processing techniques (e.g., Resize, Normalize) and augmentation techniques (e.g., RandomHorizontalFlip, RandomDistort), which were also used in our training. The first step of pre-processing involved cropping the images to the ultrasound area to eliminate regions that do not pertain directly to the ultrasound scan. The second step involved the preparation of masks containing only 5 classes (chondro-osseous border, femoral head, labrum, cartilaginous roof, and bony roof). This step was performed to create the input for Model-5 (Figure 2).

For segmentation labels from models, post-processing was used, which removed artifacts related to multiple instances of a single class caused by either small regions that needed to be cleaned or divided areas that should be connected. Moreover, mistakes for regions that were not properly marked by the model were also corrected. The best image had to be selected for post-processing. Selection was made based on a scoring algorithm that evaluated the quality of the segmentation mask and the correctness of the US image itself, whether it had all the necessary classes visible, and whether it presented a Graf standard plane.

### 2.3. Statistical Methods

The evaluation of models is crucial for understanding their effectiveness. In the field of image segmentation, one of the most widely adopted metrics is Intersection over Union (IoU). It quantifies the overlap between the predicted segmentation mask and the ground truth mask for a specific object class. Mathematically, IoU is defined as the ratio of the area of intersection between the predicted and ground truth regions to the area of their union. Formally, for a given class *C*, the *IoU* can be expressed as:

$$IoU\left(C\right)=\;\frac{\left|A\cap B\right|}{\left|A\cup B\right|}$$

where *A* represents the area of the predicted segmentation mask and *B* denotes the area of the ground truth mask. The intersection ∣*A*∩*B*∣ represents the area where both masks overlap, while the union ∣*A*∪*B*∣ represents the total area covered by at least one of the masks. A higher *IoU* value indicates better model performance. To evaluate the models, the mean Intersection over Union for a specific class across an entire dataset was computed. Another metric we used is the dice similarity coefficient (DSC), which ranges from 0 to 1. Here, *A* represents the predicted point set and *B* the labeled point set.

$$DSC(SA,SP)=\frac{2\;\cdot \;|A\;B|}{\left|A|+|B\right|}$$

All images were split into three sets: training set (for model learning), validation set (to select the best training iteration), and test set (to choose the best model among tested architectures and for final model evaluation). Images related to the same patient had to appear in only one of the sets. The split was performed in such a way that the test and validation sets each contained approximately 10% of the videos (Table 1).

Given the dependency between paired observations and non-normal distribution, the Wilcoxon signed-rank test, a non-parametric alternative to the paired *t*-test, was employed. To control for the family-wise error rate resulting from multiple comparisons, the Bonferroni correction was applied, with the significance threshold set at α = 0.01. All statistical analyses were performed using Python version 3.9.13 with the SciPy library (version 1.7.3).

## 3. Results

The dataset consisted of 375 female and 310 male US hip scans. Dysplastic hips (DDH) or physiologically immature hips (type IIa+) were diagnosed in 106 (15.47%) of all 685 DICOM files. The descriptive statistics for the dataset are presented in Table 2.

The accuracy of the model was evaluated using the IoU metric. The average value of this metric was calculated based on the test dataset for five classes (chondro-osseous border, femoral head, labrum, cartilaginous roof, and bony roof). For classes representing points (bony rim and lower limb), the Euclidean distance from the centroid of the model’s segment to the centroid of the doctor’s segment was calculated. For the baseline class, the angle of deviation between the doctor’s baseline and the model’s baseline was computed. Two models were analyzed: Model-8 (generating segments for eight classes) and Model-5 (generating segments for five classes). The analysis revealed statistically significant differences between the models in classes 1, 3, and 4. In class 1, the 8-class model demonstrated superior performance, whereas in classes 3 and 4, the 5-class model achieved higher IoU scores compared to the 8-class model. Despite reaching statistical significance, the observed effect sizes ranged from small to moderate, indicating only moderate practical relevance of these differences. The average values of the metrics calculated for the test dataset for each class are presented in Table 3.

To evaluate the effectiveness of the segmentation model, the success rate was calculated, defined as the percentage of frames in which the doctor annotated a given class and for which the Intersection over Union (IoU) between the doctor’s annotation and the model prediction reaches a value of at least 0.5 (true positives). The results are presented in Table 4.

Furthermore, for five classes (chondro-osseous border, femoral head, labrum, cartilaginous roof, and bony roof), the dice similarity coefficient on the test dataset was calculated. The average values of the metrics for each class are presented in Table 5.

The model that achieved the highest segmentation accuracy was based on the SegNeXt architecture with the MSCAN_L backbone. SegNeXt is a convolutional network that features hierarchical attention mechanisms (HAM), which contribute to its high segmentation accuracy [18]. The training parameters of our best model are detailed in Supplementary Table S2.

**Figure 3:**
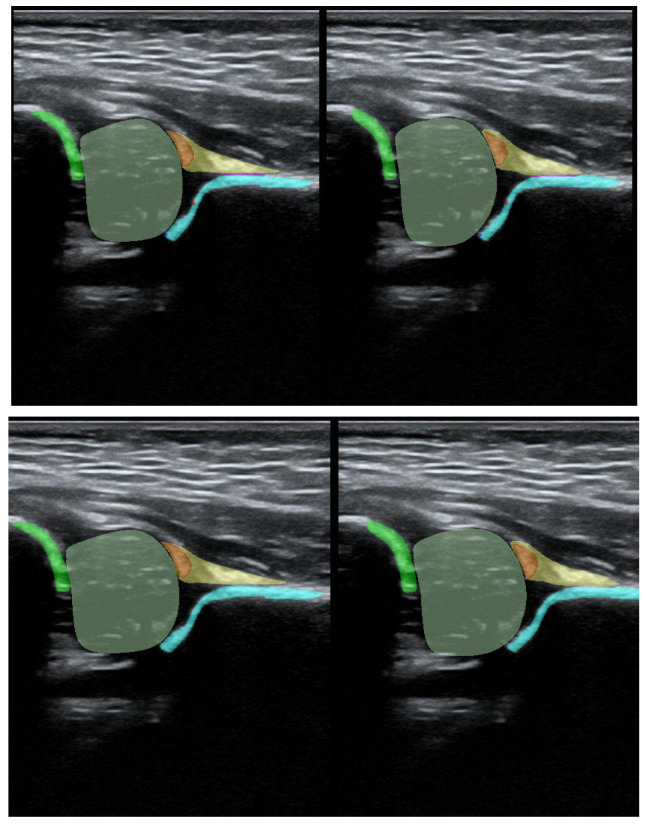
High-quality model labels (**upper**—Model-8) and (**lower**—Model-5). Annotated mask on the **left**, model mask on the **right**.

**Figure 4:**
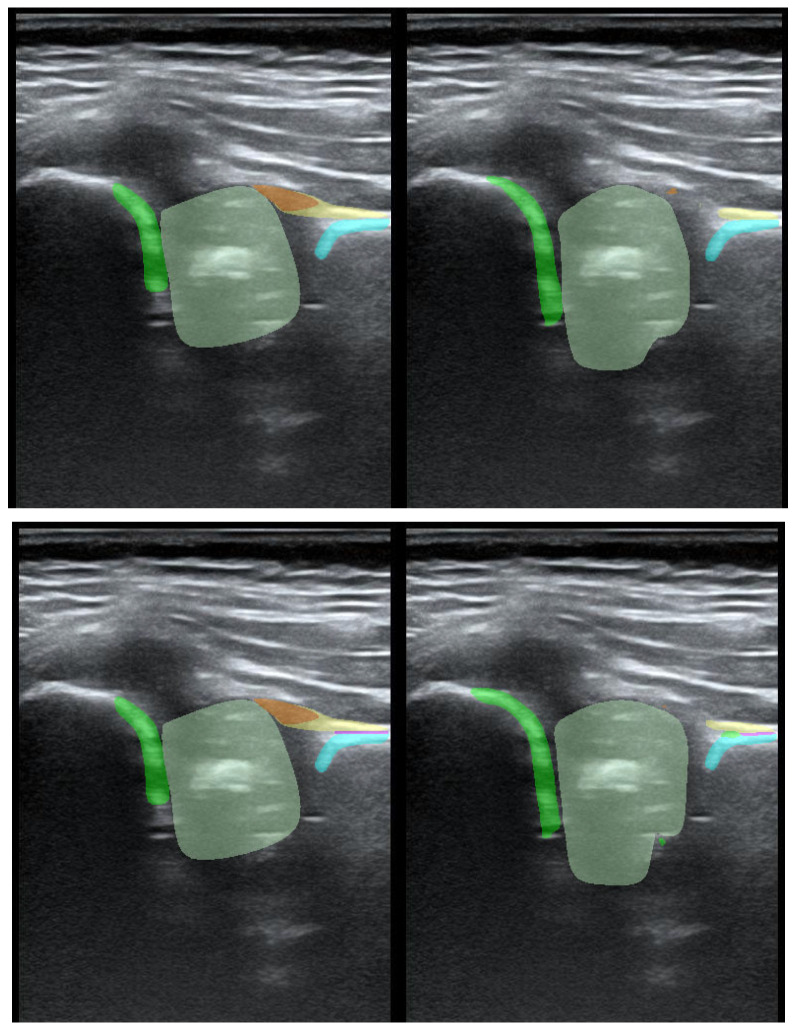
Low-quality model labels (**upper**—Model-8) and (**lower**—Model-5). Annotated mask on the **left**, model mask on the **right**.

To illustrate the performance of the segmentation model, a comparison is presented between the labels obtained by the segmentation model and the ground truth labels. Two models were analyzed: Model-8 (generating segments for eight classes) and Model-5 (generating segments for five classes). Both the cases with the highest quality model labels and the cases where the model labels were less accurate are presented. Figure 3 shows one of the best cases, in which Model-5 and Model-8 accurately labeled the classes relative to the doctor’s labels. The worst-case scenario was also analyzed, in which the prediction results differ significantly from the ground truth labels (Figure 4).

## 4. Discussion

In our work, orthopedic surgeons annotated all the structures recommended by Graf to obtain reproducible results. We expect that the structures obtained in this way can be used to make a reliable clinical diagnosis regarding the type of hip joint in the future. The SegNeXt MSCAN_L model achieved high accuracy, especially for the femoral head (IoU 0.916; DSC 0.956) and bony roof (IoU 0.725; DSC 0.841), with reliable performance for other key structures and landmarks. These results demonstrate that the system can replicate expert annotations, reduce operator dependency, and support standardized, automated DDH screening.

When compared with previous research, our approach differs in both methodology and scope, yet the achieved accuracy is consistent with or superior to reported values. Some studies, such as Quander et al. and Hareendranathan et al., use a different approach and analyze the geometry of structures instead of supervised learning methods [19]. Multiple authors analyze 3D US scans, but the methodology proposed by Graf is based on 2D US [20,21]. According to the literature, the sensitivity [95% CI] of such AI US-based systems can be as high as 0.98 [0.91–1.00]. Their specificity [95% CI] is also reported to be high—0.95 [0.81–0.99] [22].

Sezer et al. used 675 annotated US frames with “iliac wing”, “labrum”, and acetabulum. The R-CNN (Mask Region-Based Convolutional Neural Network) method was used. The authors do not report the results as an IoU metric. Instead, they report a success rate that is IoU of ≥0.5 (true positive). The average success rate was 98.25% for the iliac wing, 94.91% for the acetabulum, and 97.72% for the labrum [23]. Our success rate for the bony roof, which represents the acetabulum and iliac wing, ranged from 97.85% (Model-8) to 98.03% (Model-5). For the labrum, our model had a success rate ranging from 82.41% (Model-8) to 85.76% (Model-5).

Chen et al. described the use of an improved fully convolutional neural network to identify the femoral head on US images. The raw dataset consisted of 55 labeled US images of hip joints. The authors used the Cascaded FNet method and extracted a region of interest (ROI). The model achieved an IoU of 0.897 [24]. Our models achieved an IoU for the femoral head ranging from 0.915 (Model-5) to 0.916 (Model-8).

Li et al. used 400 US Graf standard plane images for a semi-supervised deep learning method based on a feature pyramid network (FPN) and a contrastive learning scheme based on a Siamese architecture. The annotations included the junction of cartilage and bone (chondro-osseous border), hyaline cartilage (cartilaginous roof), acetabular roof (bony roof), femoral head, joint capsule, labrum, ilium, bony part of the acetabular roof, and synovial fold. However, the authors did not include the lower limb and bony rim, which are necessary for US scan assessment according to Graf [25]. For the chondro-osseous border, the authors achieved DSC ranging from 0.685 to 0.864. Our model DSC ranged from 0.769 (Model-5) to 0.774 (Model-8). For the femoral head, the authors achieved a DSC of 0.860–0.899, and our result was 0.956 (both Model-5 and Model-8). For the labrum, the DSC in Li et al. was 0.570–0.814, and our result was 0.767 (Model-8)–0.769 (Model-5). For the cartilaginous roof, the DSC in Li et al. was 0.000–0.565, while our results were 0.799 (Model-8)–0.804 (Model-5). For the ilium and lower ilium, the authors’ results were 0.6802–0.854 and 0.379–0.819, respectively, and our result for the bony roof that combines both structures was 0.834 (Model-8)–0.841 (Model-5).

Stamper et al. was based on 190 US hip scans. The annotations included the femoral head, ilium, and labrum. U-Net was used to segment key anatomical structures. However, this study was aimed at automating femoral head coverage (FHC) calculation for DDH screening, and ours was focused on the Graf method [26]. The authors reported DSC for the femoral head of 0.924, and our result was 0.956 (both Model-8 and Model-5). The result for the ilium was 0.857, and ours for the bony roof was 0.834 (Model-8)–0.841 (Model-5). For the labrum, Stamper et al. achieved a DSC of 0.710; our result was 0.767 (Model-8) and 0.769 (Model-5).

Hu et al. used Mask R-CNN on 1231 US images of the infant hip from 632 patients and annotated the flat ilium, lower limb, labrum, CO junction (chondro-osseous border), bony rim, lower limb point, and midpoint of labrum. For the chondro-osseous border, the authors achieved a DSC ranging from 0.829 to 0.873 [27]. Our model DSC ranged from 0.769 (Model-5) to 0.774 (Model-8). For the labrum, the DSC of Hu et al. was 0.791–0.841, and our result was 0.767 (Model-8)–0.769 (Model-5). For the ilium and lower limb (marked as an area and part of the bony roof), the results were 0.869–0.869 and 0.809–0.838, respectively, and our result for the bony roof that combines both structures was 0.834 (Model-8)–0.841 (Model-5).

Golan et al. used the DCNN network and GAN. The authors chose a unique approach in which 1056 US scans of infant hips were annotated by crowdsourcing to crowdFlower platform users. The annotated structures were the ilium and acetabular roof, but the authors do not report DSC or the IoU metric [28].

Lee et al. analyzed 1243 hip US images from 168 infants. Mask annotations were made using the Computer Vision Annotation Tool (CVAT). The annotated structure was a combined area of bony roof and cartilaginous roof marked on a Graf standard plane. The authors do not report the DSC or IoU [29].

A limitation of this study is the fixed nature of the data splits and the inability to perform repeated training-validation cycles. This was a necessary methodological consequence of the project’s design, where data were acquired sequentially. Early data tranches were used for the iterative development and refinement of the pre- and post-processing algorithms. To ensure an unbiased assessment of the final model, only the last-received, entirely unseen dataset could be used for testing, precluding resampling for multiple runs or for balancing dataset ratios. Furthermore, the observed gender imbalance within the test set reflects the demographic characteristics of this final data batch and was a factor beyond our control.

The generalizability of our findings may be limited by the single-center nature of this retrospective study, as all data were obtained from one orthopedic clinic using a single ultrasound system. Differences in ultrasound equipment, examiner experience, and patient demographics across other institutions could influence model performance. At the same time, the inclusion of both universal screening and follow-up examinations reflects routine clinical practice and enhances the potential applicability of the results. To confirm robustness and broader external validity, future multicenter studies involving diverse imaging systems, operators, and populations will be required.

In our study, we developed an AI system that, for the first time, can automatically recognize all the anatomical structures required for the Graf method of hip ultrasound assessment. In practice, this means that the algorithm can “see” the same landmarks that a clinician identifies manually in order to measure the α and β angles. As a result, the calculation of these key diagnostic parameters can be performed automatically and consistently. This could reduce operator-dependent variability, help in interpreting images of lower quality, and facilitate large-scale screening programs. Importantly, the accuracy of our method, measured by IoU, DSC, and success rate, is comparable to or even better than that reported in previous studies on AI in hip ultrasound.

In future studies, we plan to design a complete CDSS class system (Clinical Decision Support System) to support doctors in their complex decision-making processes regarding DDH diagnosis and management. Recently, CDSS have seen a rapid evolution, and they are now commonly administered through computerized clinical workflows and medical records [30]. The system will be based on a machine learning (ML) algorithm that measures the α and β angles in strict accordance with the Graf method. Other data, such as patient demographics, potential risk factors for DDH, and clinical examination results, will also be collected and analyzed. Further benefits of artificial intelligence (AI) in ultrasound imaging may include improved diagnostic accuracy, leading to better patient outcomes.

## Figures and Tables

**Table 1:** Numerical and percentage share of data sets (with column percentages).

	Videos *n* (%)	Images *n* (%)
Test set	93 (13.6%)	1671 (15.5%)
Validation set	72 (10.5%)	1022 (9.5%)
Training set	520 (75.9%)	8074 (75.0%)

**Table 2:** Descriptive statistics of study population (with row percentages).

	Age, Days *	Female (%)
Test set	49 (40–75)	70 (75.3%)
Validation set	58 (40–79)	40 (55.6%)
Training set	47 (40–70)	265 (51.0%)

* Median (IQ range).

**Table 3:** Average metric values for each class for both models.

	Model-8	Model-5	*p*-Value
1. Chondro-osseous border (IoU)	0.632	0.624	<0.001
2. Femoral head (IoU)	0.916	0.915	0.26
3. Labrum (IoU)	0.621	0.625	<0.01
4. Cartilaginous roof (IoU)	0.666	0.672	<0.001
5. Bony roof (IoU)	0.716	0.725	0.89
6. Bony rim (Euclidean distance) [pixels]	4.8	-	
7. Lower limb (Euclidean distance) [pixels]	4.5	-	
8. Baseline (deflection angle) [degrees]	1.7	-	

**Table 4:** Success rate for each class for both models.

	Model-8	Model-5
Chondro-osseous border	85.88%	84.56%
Femoral head	100.00%	100.00%
Labrum	82.41%	85.76%
Cartilaginous roof	97.79%	98.50%
Bony roof	97.85%	98.03%

**Table 5:** Dice similarity coefficient for each class for both models.

	Model-8	Model-5
Chondro-osseous border	0.774	0.769
Femoral head	0.956	0.956
Labrum	0.767	0.769
Cartilaginous roof	0.799	0.804
Bony roof	0.834	0.841

## Data Availability

The data that support the findings of this study are available from Pentacomp Systemy Informatyczne S.A., but restrictions apply to the availability of these data, which were used under license for the current study, and so are not publicly available. Data are, however, available from the authors upon reasonable request and with permission of Pentacomp Systemy Informatyczne S.A.

## Supplementary Materials

The following supporting information can be downloaded at: https://www.mdpi.com/article/10.3390/jcm14176332/s1, Table S1. Architectures tested in the study; Table S2. Model training configuration.
