# Supplementary material for: Artificial Intelligence Algorithm Supporting the Diagnosis of Developmental Dysplasia of the Hip: Automated Ultrasound Image Segmentation
Source: J Clin Med. 2025 Sep 8;14(17):6332. doi: 10.3390/jcm14176332 (PMC12429232; doi:10.3390/jcm14176332)
Supplement: Supplementary file 1 [file jcm-14-06332-s001.zip › Supplementary Table 1.pdf]

Supplementary Table 1. Architectures tested in the study

SegFormer	An advanced Transformer framework designed for semantic segmentation that balances efficiency, accuracy, and robustness. The SegFormer framework consists of two main modules: a hierarchical Transformer encoder, known as a "Mix Transformer," and a lightweight All-MLP decoder. The Mix Transformer extracts both coarse and fine features using feed-forward networks (FFNs), while the All-MLP decoder directly fuses these multi-level features to predict the semantic segmentation mask. This combination allows SegFormer to achieve state-of-the-art performance in semantic segmentation, efficiently merging coarse and fine details to deliver superior accuracy while maintaining computational efficiency. SegFormer scales up to a series of models from SegFormer-B0 to SegFormer-B5.
OCRNet (Object-Contextual Representations Network)	Enhances semantic segmentation by using context from specific object regions. Unlike traditional approaches that focus on spatial positions or relational contexts between pixels, OCRNet defines soft object regions for each class using deep networks trained on ground-truth data. By collecting information from pixels within these regions, OCRNet enhances segmentation accuracy with better contextual understanding. This object-level focus sets OCRNet apart from previous methods, leading to significant improvements in segmentation quality. Semantic segmentation also involves the challenge of selecting the optimal inference resolution for different types of predictions. Fine details such as object edges benefit from higher resolutions, whereas larger structures requiring global context perform better at lower resolutions due to broader contextual observation by the network. To address this challenge effectively, a hierarchical attention mechanism has been integrated into the architecture. This mechanism predicts relative weightings between adjacent scales to refine predictions across multiple scales. To achieve superior performance in semantic segmentation, the architecture builds upon HRNet-OCR as its foundational neural network.

UNet	Frequently used in medical image segmentation, leading to the development of various UNet-based architectures. Each new version aims to address specific limitations of the original UNet and improve segmentation performance. Building on this, UNet 3+ has been proposed, which redesigns the connections between the encoder and decoder by introducing full-scale skip connections that combine detailed and semantic information to enhance segmentation accuracy. UNet 3+ effectively leverages multi-scale features, allowing it to capture both small details and overall structures in medical images, crucial for accurately identifying organ boundaries. Additionally, it employs a hybrid loss function at each output layer, which enhances the model's learning capability and performance.
U-HRNet (U-shaped High-Resolution Network)	A novel neural network architecture designed to enhance semantic representation in high-resolution tasks without increasing computational cost. It combines the strengths of two well-known networks: U-Net and HRNet. By adopting the encoder-decoder structure of U-Net, U-HRNet effectively propagates embeddings from the strongest semantic feature maps to the highest resolution feature maps. Additionally, it retains the advantages of HRNet by maintaining parallel processing of multiple scales and performing multi-scale fusion continuously. This structure supports detailed and accurate semantic representation, especially for challenging objects and large instances.
SegNeXt	Represents a pioneering approach to semantic segmentation, introducing an efficient encoder-decoder architecture that challenges traditional transformer-based models. SegNeXt innovates by prioritizing convolutional attention within its design. In the realm of semantic segmentation, where capturing fine details and global context is crucial, SegNeXt excels. The SegNeXt architecture combines innovations in convolutional neural network design for image segmentation, offering a detailed encoder-decoder framework tailored for high-performance semantic segmentation tasks. The SegNeXt architecture adopts a pyramid structure encoder, organized into four stages with

	progressively decreasing spatial resolutions. Each stage includes a Multi-Scale Convolutional Attention (MSCA) module, which replaces the self-attention mechanisms used in transformers. This strategy not only simplifies the encoding of spatial information but also outperforms both standard convolutions and self-attention mechanisms in terms of computational efficiency. The decoder component of SegNeXt is equally sophisticated, gathering multi-level features from different stages of the network. SegNeXt's decoder focuses only on features from the last three stages. This approach reduces the computational overhead associated with processing low-level information, thereby enhancing segmentation performance. The decoder structure incorporates lightweight components such as the Hamburger module to extract comprehensive global context and improve efficiency without losing accuracy. SegNeXt offers four model variants (SegNeXt-T, SegNeXt-S, SegNeXt-B, and SegNeXt-L), each differing in complexity and capacity (as indicated by parameters and layer counts). These variants allow adaptation to different computational resources.
