# Supplementary material for: Artificial Intelligence Algorithm Supporting the Diagnosis of Developmental Dysplasia of the Hip: Automated Ultrasound Image Segmentation
Source: J Clin Med. 2025 Sep 8;14(17):6332. doi: 10.3390/jcm14176332 (PMC12429232; doi:10.3390/jcm14176332)
Supplement: Supplementary file 1 [file jcm-14-06332-s001.zip › Supplementary Table 2.pdf]

Supplementary Table 2. Model training configuration.

Parameter	Description
**batch_size:** 4	The number of samples (images) in a single batch. One batch is processed in one training iteration.
**iters:** 250000	The total number of training iterations. In each iteration, the model is updated based on one batch of data.
**model:**<br>type: SegNeXt<br>**backbone:**<br>type: MSCAN_L	Parameters defining the segmentation model architecture. SegNeXt is the type of model used for image segmentation. The MSCAN_L backbone is a neural network responsible for feature extraction from the input image.
**num_classes:** 9	The number of classes in the segmentation task.
**decoder_cfg:**<br>channels: 256<br>ham_channels: 256<br>ham_kwargs: MD_R: 16<br>dropout_rate: 0.1<br>align_corners: False	Parameters configuring the model decoder, responsible for generating the final prediction maps. channels is the number of channels in the decoder layers. ham_channels refers to the number of channels in the HAM (Hierarchical Attention Mechanism) module. MD_R is the reduction ratio in the HAM module, controlling the level of feature compression. dropout_rate is the dropout coefficient, which helps prevent overfitting. align_corners is a parameter controlling whether the image edges are aligned during interpolation (e.g., during resizing).
**transforms:**<br>- type: Resize<br>target_size: [890, 1024]<br>- type: RandomHorizontalFlip<br>- type: RandomDistort<br>- type: Normalize<br>mean: [0.485, 0.456, 0.406]<br>std: [0.229, 0.224, 0.225]	A list of transformations applied to the data before being fed into the model. First, resizing the images. Second, randomly flipping the images horizontally. Third, randomly distorting the images. Finally, normalizing the images based on the mean and standard deviation for the RGB channels.

**optimizer:**<br>type: AdamW<br>weight_decay: 0.01<br>custom_cfg:<br>- name: head<br>lr_mult: 10.0<br>- name: norm<br>weight_decay_mult: 0.0	Parameters configuring the optimizer used for model training. AdamW is the type of optimizer, an extended version of Adam with regularization weights based on weight_decay. Additional parameters are set for specific model layers. The head layers have a different learning rate, while the norm layers have the regularization coefficient (weight_decay_mult) set to zero.
**lr_scheduler:**<br>type: PolynomialDecay<br>warmup_iters: 1500<br>warmup_start_lr: 1.0e-6<br>learning_rate: 0.00006<br>end_lr: 0.0<br>power: 1.0	Parameters configuring the learning rate schedule during training. PolynomialDecay is the type of schedule, which decreases the learning rate according to a polynomial decay function. warmup_iters is the number of initial iterations (warmup) during which the learning rate gradually increases from a very small value to the target value. warmup_start_lr is the initial, very low learning rate used during the warmup phase. learning_rate is the rate at which the model is trained after warmup. end_lr is the final learning rate towards which the schedule converges. power controls how fast the learning rate decreases (a value of 1.0 means linear decay).
**loss:**<br>types:<br>- type: CrossEntropyLoss<br>coef: [1]	Parameters defining the loss function. In this case, a single loss function is used (based on cross-entropy) with a weight of 1.
